# Emergency collision avoidance strategy for autonomous vehicles based on steering and differential braking

**DOI:** 10.1038/s41598-022-27296-3

**Published:** 2022-12-31

**Authors:** Haiqing Li, Taixiong Zheng, Fuhao Xia, Lina Gao, Qing Ye, Zonghuan Guo

**Affiliations:** 1 School of Advanced Manufacturing Engineering, Chongqing University of Posts and Telecommunications, Chongqing, China; 2 Chongqing Technology and Business Institute, Chongqing, 400065 China; 3 China Merchants Chongqing Communications Technology Research & Design Institute Co.,Ltd, Chongqing, China; 4 College of Automation, Chongqing University, Chongqing, China

**Keywords:** Electrical and electronic engineering, Mechanical engineering

## Abstract

This paper develops a novel integrated collision avoidance strategy for autonomous vehicles in an emergency based on steering and braking. Specifically, the framework of the collision avoidance strategy is composed of two parts: an up-level decision-making layer and a low-level controller layer. The purpose of the up-level is to select the appropriate control strategy based on the vehicle information, and the low-level is to drive the vehicle according to the instructions generated by the up-level. More concretely, a novel control strategy is proposed by integrating four-wheel steering, active rear steering, and differential braking with guaranteed path-tracking accuracy and driving stability by adaptive model predictive control (AMPC). Finally, extensive co-simulations in MATLAB/Simulink and CarSim are conducted to verify the effectiveness of the proposed collision avoidance strategy in terms of tracking error, yaw rate, and roll angle.

## Introduction

Currently, collision avoidance is becoming the standard associate system on most autonomous vehicles (AV) and is regarded as the most effective way to reduce traffic crashes, including frontal crashes, side crashes, rear-end crashes, and so on^1,2^. Collision avoidance strategy mainly includes two methods of steering and braking, which are used according to the specific traffic scenes^3^. As the initial stage of autonomous driving, advanced driving assistant systems can enhance driving safety through real-time obstacle warning and conditional intervention^4–6^. However, the ability is limited in emergency scenarios when an obstacle suddenly appears in the middle of the road^7^. For collision avoidance of AV, the main way is by designing a path planner^8^ and a tracking controller^9^ motivated for the replication of human driver’s cognition in developing motion planning and collision control^10^. Motion control for AV while performing in a collision environment is still a challenging task^11^.

The path planning techniques over the last decade for AV have been reviewed in recent articales^3,12–14^. For path tracking, active front steering (AFS)^15–17^ and active rear steering (ARS)^18,19^, are the main and common methods to enhance driving stability. However, active steering will greatly affect path tracking performance and AV also cannot safely track the planned path in an emergency^20,21^, and rollover also occurs frequently, which can cause fatal injury crashes, especially in vehicles with a high center of gravity (CG)^22,23^. Emergency steering in high-speed collision avoidance easily causes rollover due to the generation of large lateral acceleration^24^. Incorporating other actuators into the control system can improve the tracking and driving performance^23,25–27^. Moreover, the rollover performance is often ignored in tracking performance evaluation, which may cause vehicle deviation from the target path under emergency conditions^21,28,29^.

This paper proposes a novel integrated collision avoidance strategy based on steering and braking for AV in an emergency, and an up-level decision-making layer and a low-level controller layer are designed. The main contributions of this paper are summarized as follows. (1) Unlike most existing studies that investigate collision avoidance by steering or braking separately, without considering rollover stability in emergency collision avoidance. To solve this problem, we propose an integrated collision avoidance strategy by coordinating the tracking performance, yaw stability, and rollover stability based on active steering and braking. (2) To ensure the tracking accuracy and yaw stability in collision avoidance, we designed an up-level decision-making layer and a low-level controller layer by integrating with four-wheel steering and active rear steering, meanwhile, the weight coefficient matrix on path tracking and stability responses are analyzed based on AMPC. (3) To guarantee rollover stability in emergency collision avoidance, a priority weight will be given to the yaw and roll aspect even though the path tracking performance became worse. The effectiveness and superiority of our method are tested by simulation under different control strategies.

The rest of this article is organized as follows. “Vehicle dynamic model” section describes the vehicle models of AMPC. The structure of the integrated emergency collision avoidance strategy by 4 WS, ARS and DB based on AMPC theory is set up in “Collision avoidance strategy design” section. “Simulation results” section, verifies the effectiveness and superiority of the designed integrated collision avoidance strategy. “Conclusions and outlooks” section, summarizes the conclusions and future directions.

### Vehicle dynamic model

**Figure 1:**
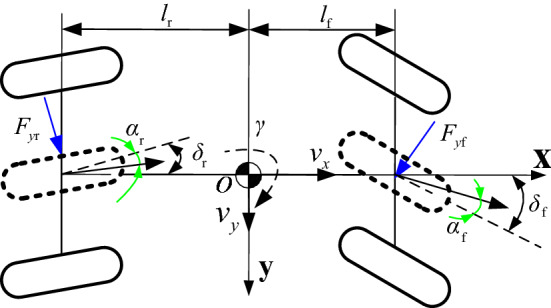
Two DOF vehicle dynamic models.

The two DOF vehicle model is taken as the basis for the design of the steering control. Figure 1 describes the vehicle dynamic characteristics of the two DOF models.

Assuming the vehicle runs in ideal conditions, the vehicle’s lateral and yaw motion dynamic equations can be expressed, respectively, as^16^

1 $$ m\left( {\dot{v}_{y} + v_{x} \gamma } \right) = F_{{y{\text{f}}}} \cos \delta_{{\text{f}}} + F_{{y{\text{r}}}} \cos \delta_{{\text{r}}} $$

2 $$ I_{z} \dot{\gamma } = l_{{\text{f}}} F_{{y{\text{f}}}} \cos \delta_{{\text{f}}} - l_{{\text{r}}} F_{{y{\text{r}}}} \cos \delta_{{\text{r}}} $$

where *m* is the mass of the vehicle; *v*_*x*_ and *v*_*y*_ are the longitudinal and lateral velocities; *l*_f_ and *l*_r_ are the distances from CG to the front and rear axles, respectively; *F*_*y*f_ and *F*_*y*r_ are the vehicle’s lateral forces; *δ*_f_ and *δ*_r_ are the steering angle of the front and rear wheels; I_z_ is the yaw moment of inertia.

The slip angle of the front and rear tires is obtained as

3 $$ \left\{ \begin{gathered} \alpha_{{\text{f}}} = \delta_{{\text{f}}} - \frac{{v_{y} + l_{{\text{f}}} \gamma }}{{v_{x} }} \hfill \\ \alpha_{{\text{r}}} = \delta_{{\text{r}}} - \frac{{v_{y} - l_{{\text{r}}} \gamma }}{{v_{x} }} \hfill \\ \end{gathered} \right. $$

Then, the linear tire model can be expressed as

4 $$ F_{{y{\text{f}}}} = k_{{\text{f}}} \alpha_{{\text{f}}} $$

5 $$ F_{{y{\text{r}}}} = k_{{\text{r}}} \alpha_{{\text{r}}} $$

where *k*_f_, *k*_r_ are the vehicle cornering stiffness. Then the vehicle dynamic equations are obtained as

6 $$ \left\{ \begin{gathered} m\dot{v}_{y} { + }\frac{{\left( {k_{{\text{f}}} { + }k_{{\text{r}}} } \right)v_{y} }}{{v_{x} }}{ + }\left( {mv_{x} { + }\frac{{l_{{\text{f}}} k_{{\text{f}}} - l_{r} k_{{\text{r}}} }}{{v_{x} }}} \right) \cdot \gamma = k_{{\text{f}}} \delta_{{\text{f}}} + k_{{\text{r}}} \delta_{{\text{r}}} \hfill \\ I_{z} \dot{\gamma } + \frac{{\left( {l_{{\text{f}}}^{{2}} k_{{\text{f}}} + l_{{\text{r}}}^{{2}} k_{{\text{r}}} } \right)\gamma }}{{v_{x} }} + \frac{{\left( {l_{{\text{f}}} k_{{\text{f}}} - l_{{\text{r}}} k_{{\text{r}}} } \right)v_{y} }}{{v_{x} }} = l_{{\text{f}}} k_{{\text{f}}} \delta_{{\text{f}}} - l_{{\text{r}}} k_{{\text{r}}} \delta_{{\text{r}}} \hfill \\ \end{gathered} \right. $$

Written in the state equation of Eq. (6), as

7 $$ {\dot{\mathbf{x}}} = {\mathbf{Ax}} + {\mathbf{Bu}} $$

where **x** = [*v*_*y*_, *γ*], **u** = [*δ*_f_, *δ*_r_]. Additionally,

$${\mathbf{A}}{ = } - \frac{1}{{v_{x} }}\left[ \begin{gathered} \frac{1}{m}\left( {k_{{\text{f}}} { + }k_{{\text{r}}} } \right)\;\;\;\;\frac{1}{m}\left( {l_{{\text{f}}} k_{{\text{f}}} - l_{r} k_{{\text{r}}} } \right) + v_{x}^{2} \hfill \\ \frac{{l_{{\text{f}}} k_{{\text{f}}} - l_{{\text{r}}} k_{{\text{r}}} }}{{I_{z} }}\;\;\;\;\;\;\;\;\;\;\frac{{l_{{\text{f}}}^{{2}} k_{{\text{f}}} + l_{{\text{r}}}^{{2}} k_{{\text{r}}} }}{{I_{z} }} \hfill \\ \end{gathered} \right]{, }{\mathbf{B}}{ = }\left[ \begin{gathered} \frac{1}{m}k_{{\text{f}}} \;\;\;\frac{1}{m}k_{{\text{r}}} \hfill \\ \frac{{l_{{\text{f}}} k_{{\text{f}}} }}{{I_{z} }}\;\;\; - \frac{{l_{{\text{r}}} k_{{\text{r}}} }}{{I_{z} }} \hfill \\ \end{gathered} \right]$$.

Finding the characteristic roots of Eq. (7), can be written as

8 $$ \det (s{\mathbf{I}} - {\mathbf{A}}) = \left| \begin{gathered} s{ + }\frac{1}{{mv_{x} }}\left( {k_{{\text{f}}} { + }k_{{\text{r}}} } \right)\;\;\;v_{x} + \frac{1}{{mv_{x} }}\left( {l_{{\text{f}}} k_{{\text{f}}} - l_{r} k_{{\text{r}}} } \right) \hfill \\ \frac{1}{{I_{z} v_{x} }}\left( {l_{{\text{f}}} k_{{\text{f}}} - l_{{\text{r}}} k_{{\text{r}}} } \right)\;\;\;s{ + }\frac{1}{{I_{z} v_{x} }}\left( {l_{{\text{f}}}^{{2}} k_{{\text{f}}} + l_{{\text{r}}}^{{2}} k_{{\text{r}}} } \right) \hfill \\ \end{gathered} \right| = 0 $$

9 $$ \begin{aligned} & \Rightarrow  s^{2} + \left( {\frac{{l_{{\text{f}}}^{{2}} k_{{\text{f}}} + l_{{\text{r}}}^{{2}} k_{{\text{r}}} }}{{I_{z} v_{x} }} + \frac{{k_{{\text{f}}} { + }k_{{\text{r}}} }}{{mv_{x} }}} \right)s + \frac{{k_{{\text{f}}} { + }k_{{\text{r}}} }}{{mv_{x} }} \cdot \frac{{l_{{\text{f}}}^{{2}} k_{{\text{f}}} + l_{{\text{r}}}^{{2}} k_{{\text{r}}} }}{{I_{z} v_{x} }} \\&\quad { - }\frac{{l_{{\text{f}}} k_{{\text{f}}} - l_{{\text{r}}} k_{{\text{r}}} }}{{I_{z} v_{x} }} \cdot \left( {\frac{{l_{{\text{f}}} k_{{\text{f}}} - l_{r} k_{{\text{r}}} }}{{mv_{x} }} + v_{x} } \right)A\; = 0\; \\ \end{aligned} $$

10 $$ \Rightarrow s^{2} + \left( {\frac{{l_{{\text{f}}}^{{2}} k_{{\text{f}}} + l_{{\text{r}}}^{{2}} k_{{\text{r}}} }}{{I_{z} v_{x} }} + \frac{{k_{{\text{f}}} { + }k_{{\text{r}}} }}{{mv_{x} }}} \right)s + \frac{{l^{2} k_{{\text{f}}} k_{{\text{r}}} }}{{mI_{z} v_{x}^{2} }}\left( {1 - \frac{{m\left( {l_{{\text{f}}} k_{{\text{f}}} - l_{r} k_{{\text{r}}} } \right)}}{{l^{2} k_{{\text{f}}} k_{{\text{r}}} }}v_{x}^{2} } \right)\;\;{ = }0 $$

11 $$ \Rightarrow s^{2} + \left( {\frac{{l_{{\text{f}}}^{{2}} k_{{\text{f}}} + l_{{\text{r}}}^{{2}} k_{{\text{r}}} }}{{I_{z} v_{x} }} + \frac{{k_{{\text{f}}} { + }k_{{\text{r}}} }}{{mv_{x} }}} \right)s + \frac{{l^{2} k_{{\text{f}}} k_{{\text{r}}} }}{{mI_{z} v_{x}^{2} }}\left( {1{ + }Kv_{x}^{2} } \right)\;{ = }0 $$

where, $K = \frac{{m\left( {l_{{\text{r}}} k_{{\text{r}}} - l_{{\text{f}}} k_{{\text{f}}} } \right)}}{{l^{2} k_{{\text{f}}} k_{{\text{r}}} }}$. It can be concluded from Eq. (11) that the 4 WS vehicle is unstable if $K > - \frac{1}{{v_{x}^{2} }}$.

In stationary situations, $\dot{v}_{y}$ = 0, $\dot{\gamma }$ = 0. Equation (6) can be expressed as

12 $$ \left[ {mv_{x}^{2} \left( {l_{{\text{f}}} k_{{\text{f}}} - l_{{\text{r}}} k_{{\text{r}}} } \right) - l^{2} k_{{\text{f}}} k_{{\text{r}}} } \right]\gamma \; = - k_{{\text{f}}} k_{{\text{r}}} lv_{x} \left( {\delta_{{\text{f}}} - \delta_{{\text{r}}} } \right) $$

13 $$ \Rightarrow \left( {1{ + }Kv_{x}^{2} } \right)\gamma \; = \frac{{v_{x} }}{l}\left( {\delta_{{\text{f}}} - \delta_{{\text{r}}} } \right) $$

14 $$ \Rightarrow \delta_{{\text{f}}} - \delta_{{\text{r}}} = \frac{\gamma l}{{v_{x} }}\left( {1{ + }Kv_{x}^{2} } \right) $$

It has been proven that the lateral stability evaluation index of *γ* is adjustable by controlling *δ*_r_ before a vehicle reaches the limit operating condition^22^. According to Eq. (14), the rear steering can be designed as

15 $$ \delta_{{\text{r}}} = \delta_{{\text{f}}} - \frac{{\left( {1{ + }Kv_{x}^{2} } \right)l}}{{v_{x} }} \cdot \gamma $$

**Figure 2:**
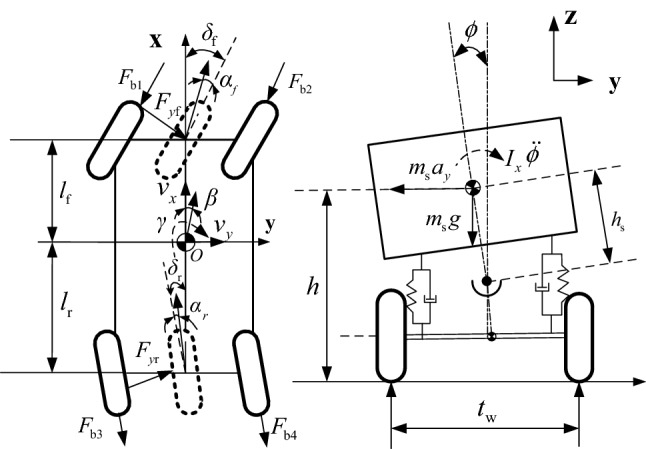
Four DOF linear vehicle mode.

The Four DOF vehicle model is taken as the basis for the design of differential braking for rollover control, which is given in Fig. 2.

The equations of body motion for the four DOF model can be described as^26^

16 $$ m\left( {\dot{v}_{x} - \beta v_{x} \gamma } \right) = \left( {F_{{{\text{b1}}}} { + }F_{{{\text{b2}}}} } \right)\cos \delta_{{\text{f}}} + \left( {F_{{{\text{b3}}}} { + }F_{{{\text{b4}}}} } \right)\cos \delta_{{\text{r}}} $$

17 $$ I_{z} \dot{\gamma } = l_{{\text{f}}} F_{{y{\text{f}}}} \cos \delta_{{\text{f}}} - l_{{\text{r}}} F_{{y{\text{r}}}} \cos \delta_{{\text{r}}} - \left( {F_{{{\text{b1}}}} + F_{{{\text{b3}}}} - F_{{{\text{b2}}}} - F_{{{\text{b4}}}} } \right)t_{{\text{w}}} /2 $$

18 $$ mv_{x} (\gamma + \dot{\beta }) = F_{{y{\text{f}}}} \cos \delta_{{\text{f}}} + F_{{y{\text{r}}}} \cos \delta_{{\text{r}}} $$

19 $$ I_{x} \dot{p} = m_{{\text{s}}} a_{y} h_{{\text{s}}} - C_{\phi } p{ + }m_{s} gh_{s} \phi - (K_{{\phi {\text{f}}}} + K_{{\phi {\text{r}}}} )\phi $$

Considering Eqs. (16–19), the dynamic equations of the vehicle can be rewritten as

20 $$ \dot{x} = f\left( x \right) + Nu $$

where, the state variables $x = \left[ {v_{x} \;\gamma \;\;\beta \;\;\phi \;\;p} \right]^{{\text{T}}}$, the inputs $u = \left[ {F_{{{\text{b1}}}} \;F_{{{\text{b2}}}} \;F_{{{\text{b3}}}} \;F_{{{\text{b4}}}} } \right]$,

$$ \begin{gathered} f\left( x \right) = \left[ \begin{gathered} \beta v_{x} \gamma \hfill \\ \frac{{l_{f} F_{yf} \cos \delta_{f} - l_{r} F_{yr} \cos \delta_{{\text{r}}} }}{{I_{z} }} \hfill \\ \frac{{F_{{y{\text{f}}}} \cos \delta_{f} + F_{{y{\text{r}}}} \cos \delta_{{\text{r}}} }}{{mv_{x} }} - \gamma \hfill \\ p \hfill \\ \frac{{\left( {F_{{y{\text{f}}}} \cos \delta_{{\text{f}}} + F_{{y{\text{r}}}} \cos \delta_{{\text{r}}} } \right)h_{s} - C_{\phi } p{ + }m_{{\text{s}}} gh_{{\text{s}}} \phi - (K_{{\phi {\text{f}}}} + K_{{\phi {\text{r}}}} )\phi }}{{I_{x} }} \hfill \\ \end{gathered} \right],\;N = \left[ \begin{gathered} \frac{{\cos \delta_{{\text{f}}} }}{m}\;\;\;\;\;\frac{{\cos \delta_{{\text{f}}} }}{m}\;\;\;\;\;\;\frac{1}{m}\;\;\;\;\;\;\;\;\;\frac{1}{m}\;\;\; \hfill \\ - \frac{{t_{{\text{w}}} }}{{2I_{z} }}\;\;\;\;\;\;\;\;\frac{{t_{{\text{w}}} }}{{2I_{z} }}\;\;\;\;\; - \frac{{t_{{\text{w}}} }}{{2I_{z} }}\;\;\;\;\;\;\frac{{t_{{\text{w}}} }}{{2I_{z} }} \hfill \\ \;\;\;0\;\;\;\;\;\;\;\;\;\;\;\;0\;\;\;\;\;\;\;\;\;\;0\;\;\;\;\;\;\;\;\;\;\;0 \hfill \\ \;\;\;0\;\;\;\;\;\;\;\;\;\;\;\;0\;\;\;\;\;\;\;\;\;\;0\;\;\;\;\;\;\;\;\;\;\;0 \hfill \\ \;\;\;0\;\;\;\;\;\;\;\;\;\;\;\;0\;\;\;\;\;\;\;\;\;\;0\;\;\;\;\;\;\;\;\;\;\;0 \hfill \\ \hfill \\ \end{gathered} \right] \hfill \\ \hfill \\ \end{gathered} $$

### Collision avoidance strategy design

**Figure 3:**
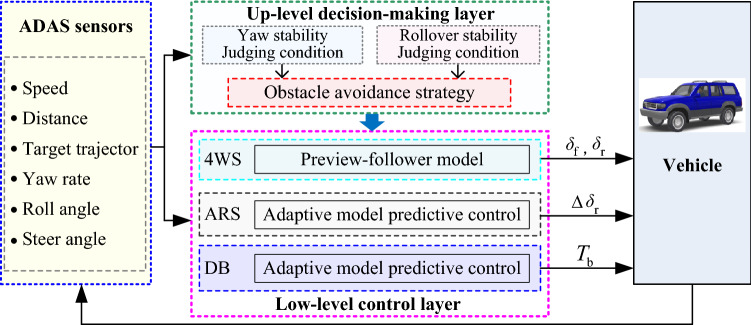
General framework of emergency collision avoidance strategy.

The integrated collision avoidance strategy is composed of two parts: an up-level decision-making layer and a low-level controller layer. The purpose of the up-level is to select the appropriate control strategy based on the ADAS sensors, and the low-level is to control the vehicle according to the instructions generated by the up-level. Considering both path-tracking accuracy and driving stability, the collision avoidance controller is proposed by integrating with 4 WS, ARS, and DB based on AMPC. The general framework of the collision avoidance strategy is illustrated in Fig. 3.

### Collision avoidance by steering control

#### General 4 WS

**Figure 4:**
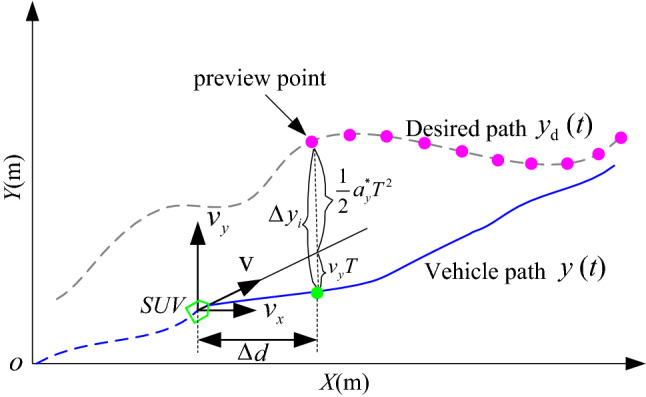
Preview-follower control.

A ten-point preview-follower control model is designed in Fig. 4.

The preview distance is ∆*d*. The error of the lateral position between the desired path and the vehicle path can be defined as^19^

21 $$ \Delta y_{i} = y_{{{\text{d}}i}} (t{ + }T) - y_{i} (t) $$

22 $$ T = {{\Delta d} \mathord{\left/ {\vphantom {{\Delta d} {v_{x} }}} \right. \kern-0pt} {v_{x} }} $$

where $y_{{{\text{di}}}} \left( t \right)$ and $y_{i} \left( t \right)$ are the desired and actual lateral displacement, *T* is the preview time, and *T* = 1 s. It presumes that the tracking error ∆*y*_*i*_ can be eliminated after *T*. Thus,

23 $$ y_{{\text{d}}} (t){ = }y(t{ + }T) + \Delta y_{i}^{*} $$

24 $$ \Rightarrow \Delta y_{i}^{ * } = y(t{ + }T) - y_{{\text{d}}} (t){ = }\dot{y}{(}t{)}T + \frac{{a_{y}^{ * } T^{2} }}{2} $$

then

25 $$ a_{y}^{ * } = \frac{{2\left( {y(t{ + }T) - y(t) - \dot{y}{(}t{)}T} \right)}}{{T^{2} }} $$

where, $a_{y}^{ * }$ is the ideal value of $a_{y}^{{}}$.

The realistic absolute value of *v*_*y*_ $\ll$ *v*_*x*_. Thus, the total velocity **v** = $\sqrt {v_{x}^{2} + v_{y}^{2} }$≈ *v*_*x*_. Since v = *γR* (vehicle turning radius), then,

26 $$ a_{y} = \gamma^{2} R = \left( {\frac{{v_{x} }}{R}} \right)^{2} R = \gamma \left( {\frac{{v_{x} }}{R}} \right)R = \gamma v_{x} $$

Substituting Eq. (26) to (15), the 4 WS system is designed as

27 $$ \delta_{{\text{f}}} - \delta_{{\text{r}}} = \frac{{\left( {1{ + }Kv_{x}^{2} } \right)l}}{{v_{x}^{2} }}a_{y} $$

To achieve $a_{y}^{ * }$ for 4 WS,$\delta_{{\text{f}}}^{ * }$ and $\delta_{{\text{r}}}^{ * }$ should be applied as

28 $$ \delta_{{\text{f}}}^{ * } - \delta_{{\text{r}}}^{ * } = \frac{{a_{y}^{ * } }}{{G_{{{\text{a}}y}} }} $$

where

29 $$ G_{{{\text{a}}y}}^{{}} = \frac{{v_{x}^{2} }}{{l\left( {1 + Kv_{x}^{2} } \right)}} $$

**Figure 5:**
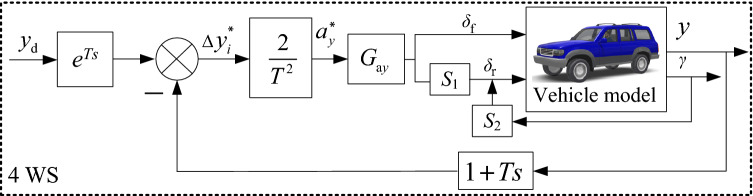
4 WS using preview-follower.

The control architecture of the 4 WS by preview-follower is illustrated in Fig. 5.

#### MPC design

For the linear vehicle model, the global y position:

30 $$ \dot{Y} = v_{x} \psi + v_{y} $$

where *ψ* is the yaw angle.

The relevant equation of state is^28^:

31 $$ \dot{x} = Ax + Bu $$

where, *x* = [*y*` *ψ γ Y*], *u* = [*δ*_f_, *δ*_r_],

$$ A{ = }\left[ \begin{gathered} \frac{ - 1}{{mv_{x} }}\left( {k_{{\text{f}}} { + }k_{{\text{r}}} } \right)\;\;\;\;0\;\;\; - \frac{{l_{{\text{f}}} k_{{\text{f}}} - l_{r} k_{{\text{r}}} }}{{mv_{x} }} - v_{x} \;\;\;\;0 \hfill \\ \;\;\;\;\;\;\;0\;\;\;\;\;\;\;\;\;\;\;\;0\;\;\;\;\;\;\;\;\;\;\;\;\;\;\;1\;\;\;\;\;\;\;\;\;\;\;\;\;\;0 \hfill \\ - \frac{{l_{{\text{f}}} k_{{\text{f}}} - l_{{\text{r}}} k_{{\text{r}}} }}{{I_{z} v_{x} }}\;\;\;\;\;0\;\;\;\; - \;\frac{{l_{{\text{f}}}^{{2}} k_{{\text{f}}} + l_{{\text{r}}}^{{2}} k_{{\text{r}}} }}{{I_{z} v_{x} }}\;\;\;\;\;\;\;\;0 \hfill \\ \;\;\;\;\;\;\;\;1\;\;\;\;\;\;\;\;\;\;\;\;v_{x} \;\;\;\;\;\;\;\;\;\;\;\;\;0\;\;\;\;\;\;\;\;\;\;\;\;\;\;0 \hfill \\ \end{gathered} \right]{,}\;B{ = }\left[ \begin{gathered} \frac{{k_{{\text{f}}} }}{m}\;\;\;\;0\;\;\;\;\;\frac{{k_{{\text{r}}} }}{m}\;\;\;\;\;0 \hfill \\ \frac{{l_{{\text{f}}} k_{{\text{f}}} }}{{I_{z} }}\;\;0\;\;\; - \frac{{l_{{\text{r}}} k_{{\text{r}}} }}{{I_{z} }}\;\;\;0 \hfill \\ \end{gathered} \right] $$

The discrete state space of Eq. (31) is achieved based on the forward Euler method, as

32 $$ \dot{x} = \frac{1}{T}\left( {x\left( {k + 1} \right) - x\left( k \right)} \right) = Ax\left( k \right) + Bu\left( k \right) $$

33 $$ \Rightarrow x\left( {k + 1} \right) = \left( {TA{\text{ + I}}} \right)x\left( k \right) + TBu\left( k \right) $$

34 $$ \Rightarrow x\left( {k + 1} \right) = \tilde{A} \cdot x\left( k \right) + \tilde{B} \cdot u\left( k \right) $$

where, $\tilde{A}{ = }TA{\text{ + I,}}\;\;\tilde{B}{ = }TB$ and *x*(*k*) are the vehicle states at time *k*; *x*(*k* + 1) are the vehicle states at time *k* + 1; **I** is a unit matrix; *T* is the discretization time.

A unique feature of the MPC method is that it can forecast the system’s future state. The predicted state within the *P* control cycle as^30^:

35 $$ X_{k} = \left[ {x\left( {k + 1\left| k \right.} \right)^{T} ,x\left( {k + 2\left| k \right.} \right)^{T} , \cdots ,x\left( {k + N_{p} \left| k \right.} \right)^{T} } \right]^{T} $$

36 $$ U_{k} = \left[ {u\left( {k\left| k \right.} \right)^{T} ,u\left( {k + 1\left| k \right.} \right)^{T} , \cdots ,u\left( {k + N_{c} - 1\left| k \right.} \right)^{T} } \right]^{T} $$

where *x*(*k* + 1|*k*) and *u*(*k* + 1|*k*) are the states predicted at time *k* + 1 computed at time *k*, *N*_*p*_ is the predictive step length, and *N*_*c*_ is the control step length.

The system states of the future *P* control periods are predicted by discretization of the state equations as:

37 $$ x\left( {k + 1\left| k \right.} \right) = \tilde{A} \cdot x\left( k \right) + \tilde{B} \cdot u\left( {k\left| k \right.} \right) $$

38 $$ x\left( {k + 2\left| k \right.} \right) = \tilde{A}^{2} \cdot x\left( k \right) + \tilde{A}\tilde{B} \cdot u\left( {k\left| k \right.} \right) + \tilde{B} \cdot u\left( {k + 1\left| k \right.} \right) $$

39 $$ x\left( {k + 3\left| k \right.} \right) = \tilde{A}^{3} \cdot x\left( k \right) + \tilde{A}^{2} \tilde{B} \cdot u\left( {k\left| k \right.} \right) + \tilde{A}\tilde{B} \cdot u\left( {k + 1\left| k \right.} \right) + \tilde{B} \cdot u\left( {k + 2\left| k \right.} \right) $$

40 $$ x\left( {k + P\left| k \right.} \right) = \tilde{A}^{P} \cdot x\left( k \right) + \sum\limits_{i = 0}^{P - 1} {\tilde{A}^{P - 1 - i} } \tilde{B} \cdot u\left( {k + i\left| k \right.} \right) $$

Written in state matrix form, then

41 $$ X_{k} = \phi x\left( k \right){ + }\varphi U_{k} $$

where, $$\phi = \left[ {\tilde{A},\;\tilde{A}^{2} \cdots \tilde{A}^{P} } \right]^{T} ,\;\varphi = \left[ \begin{gathered} \tilde{A}^{1 - 1} \tilde{B}\;\;\;\; \cdots \;\;\;\;\;\;\;0\;\;\;\;\;\;\;0 \hfill \\ \tilde{A}^{2 - 1} \tilde{B}\;\;\tilde{A}^{2 - 2} \tilde{B}\;\;\; \cdots \;\;\;\;\;\;\;0\; \hfill \\ \;\;\; \vdots \;\;\;\;\;\;\;\;\; \vdots \;\;\;\;\;\;\; \ddots \;\;\;\;\;\;\; \vdots \hfill \\ \tilde{A}^{P - 1} \tilde{B}\;\;\tilde{A}^{P - 2} \tilde{B}\;\; \cdots \;\;\tilde{A}^{P - P} \tilde{B}\; \hfill \\ \end{gathered} \right]$$.

Define a sequence of reference values in the predicted *P* time as^30^:

42 $$ R_{k} = \left[ {r_{{{\text{ref}}}} \left( {k + 1} \right)^{T} ,r_{{{\text{ref}}}} \left( {k + 2} \right)^{T} , \cdots ,r_{{{\text{ref}}}} \left( {k + P} \right)^{T} } \right]^{T} $$

where, $$r_{{{\text{ref}}}} = [Y_{{{\text{ref}}}} ,\gamma_{{{\text{ref}}}} ]$$.

According to the cumulative error between the predicted state vector and the reference value, the optimization objective function considering the constraints is as follows:

43 $$ J(U_{k} ) = \sum\limits_{i = 1}^{{N_{p} }} {||X_{k} - R_{k} } ||_{Q}^{2} + \sum\limits_{i = 0}^{{N_{c - 1} }} {\left| {U_{k} } \right|_{R}^{2} } $$

where *Q* and *R* are the weight matrices.

The MPC is an optimal control method. Combining Eqs. (41–43), the optimization problems can be solved for the active rear steering controller as

44 $$ \mathop {{\text{min}}}\limits_{\Delta u,\varepsilon } \{ J(x(t),\;u(t - 1),\;\Delta u(t))\}  $$

Subject to: $$x\left( {k + 1} \right) = \tilde{A} \cdot x\left( k \right) + \tilde{B} \cdot u\left( k \right)$$

$$ \begin{array}{*{20}c} {} & {u_{\min } (k)} \\ \end{array} \le u_{t} (k) \le u_{\max } (k) $$

$$ \Delta u_{\min } (k) \le \Delta u_{t} (k) \le \Delta u_{\max } (k) $$

$$ a_{y,\min } - \varepsilon \le a_{y} \le a_{y,\min } + \varepsilon $$

45 $$ \varepsilon > 0 $$

where, $\left| {a_{y} } \right| \le ug$, *u* is the Road adhesion coefficient.

#### ARS using AMPC design

Through a rolling optimization strategy, the MPC can not only address the issues of tracking capability and uncertain parameters but also ensure driving stability.

*Remark 1*: According to 4WS, the rear steering angle by ARS + MPC is calculated as $\delta_{{\text{r}}}^{^{\prime}} = \delta_{{\text{r}}} + \Delta \delta_{{\text{r}}}$ .

In the MPC controller, it was assumed that the vehicle was driving at a constant speed. The vehicle dynamics do not change, and **A** (state matrix) is constant. However, if the longitudinal speed varies as the vehicle travels, **A** also changes.

*Remark 2*: Considering conventional MPC cannot handle the nonlinear dynamics because it employs a fixed interior vehicle model. Thus, the AMPC (a combination of MPC and online update of the model parameters) is designed to address the changing vehicle dynamics as $v_{x} (k + 1) = v_{x} (k)$, k = 1,2,3

**Figure 6:**
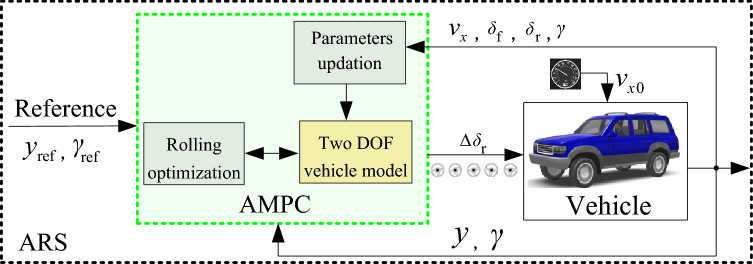
ARS using AMPC.

Figure 6 shows the flow chart of the ARS by AMPC.

The prebuilt function of the AMPC takes *v*_*x*_, steering angle, and vehicle state of *γ* as inputs. For the design of ARS, the reference path *y*_ref_ is generated by the path planning model and the reference yaw rate *γ*_ref_ can be obtained as^26^:

46 $$ \gamma_{{{\text{ref}}}} = \min \left\{ {\frac{{v_{x} }}{{l\left( {1 + Kv_{x}^{2} } \right)}} \cdot \delta_{{\text{f}}} \;,\;\frac{\mu g}{{v_{x} }}} \right\} $$

To make the vehicle path tracking controller fit for emergency collision avoidance, the preferred weights will be applied to the stability aspect, and ARS based on AMPC will be used even though the path tracking performance worsens.

### Collision avoidance by braking control

For SUV vehicles, a rollover may still occur even with the 4 WS + ARS system on account of the high CG in emergency collision avoidance. For rollover control, a rollover prediction module must be designed. Load transfer ratio (LTR) is commonly used as

47 $$ LTR = \frac{{F_{{z{\text{r}}}} - F_{{z{\text{l}}}} }}{{F_{{z{\text{r}}}} + F_{{z{\text{l}}}} }} $$

where, $F_{{z{\text{r}}}} ,F_{{z{\text{l}}}}$ are right, left vertical loads on the wheel.

By analyzing the vehicle mechanism of roll, the LTR is rewritten as^27^:

48 $$ LTR{ = }\frac{{2C_{\phi } p{ + 2}(K_{{\phi {\text{f}}}} + K_{{\phi {\text{r}}}} )\phi }}{{mgt_{{\text{w}}} }} $$

If |LTR| is larger than 0.8, it means the vehicles are in grave danger of rollover. Therefore, the threshold value of LTR_S_ = 0.8. When the rollover is about to occur, the braking instruction is ordered for rollover control, however, the brake force may be too large, and to prevent wheels from locking, the ABS controller^31^ is also added.

To obtain the braking force as |LTR|> LTR_S_, an AMPC is used to calculate the braking torque. The 4-DOF vehicle model is taken as the basis for the rollover controller. Then, its discrete and incremental form of Eq. (20) is represented as:

49 $$ \Delta x\left( {k + 1} \right) = A^{\prime} \cdot \Delta x\left( k \right) + B^{\prime} \cdot \Delta u\left( k \right) $$

where, $$A^{\prime}{\text{ = I + }}T \cdot \frac{\partial f}{{\partial x}}\left| {_{x\left( k \right)} } \right.{,}\;\;B^{\prime}{ = }TN$$.

The controlled output *X’*_*k*_ is defined as yaw rate *γ* and LTR.

50 $$ X^{\prime}_{{}} { = }\left[ {\gamma \;\;{\text{LTR}}} \right]^{{\text{T}}} { = }C^{\prime}x $$

where, $$C^{\prime} = \left[ \begin{gathered} 0\;\;\;\;\;1\;\;\;\;\;\;0\;\;\;\;\;\;\;\;\;\;\;0\;\;\;\;\;\;\;\;\;\;\;\;0\;\;\; \hfill \\ 0\;\;\;\;\;0\;\;\;\;\;0\;\;\;\frac{{2(K_{{\phi {\text{f}}}} + K_{{\phi {\text{r}}}} )}}{{mgt_{w} }}\;\;\;\frac{{2C_{\phi } }}{{mgt_{w} }} \hfill \\ \end{gathered} \right]$$.

Considering the actuator’s ability, the input of the AMPC controller should satisfy

51 $$ \begin{array}{*{20}c} {} & {u^{\prime}_{\min } (k)} \\ \end{array} \le u^{\prime}_{t} (k) \le u^{\prime}_{\max } (k) $$

The output needs to follow the references and minimize the input simultaneously. Thus, the AMPC cost function is designed as

52 $$ J^{\prime}(U_{k} ) = \sum\limits_{i = 1}^{{N_{p} }} {||X^{\prime}_{k} - R^{\prime}_{k} } ||_{Q}^{2} + \sum\limits_{i = 0}^{{N_{c - 1} }} {\left| {U^{\prime}_{k} } \right|_{R}^{2} } $$

The optimization problems can be solved for the active controller as.

$$\mathop {{\text{min}}}\limits_{{\Delta u^{\prime},\varepsilon }} \{ J(x(t),\;u(t - 1),\;\Delta u(t),\;N_{p} {,}N_{c} )\} $$,

Subject to: $$x\left( {k + 1} \right) = \tilde{A}^{\prime}x\left( k \right) + \tilde{B}^{\prime}u\left( k \right)$$,

$$\begin{array}{*{20}c} {} & {u^{\prime}_{\min } (k)} \\ \end{array} \le u^{\prime}_{t} (k) \le u^{\prime}_{\max } (k)$$,

$$\Delta u^{\prime}_{\min } (k) \le \Delta u^{\prime}_{t} (k) \le \Delta u^{\prime}_{\max } (k)$$,

$$a_{x,\min } - \varepsilon \le a_{x} \le a_{x,\min } + \varepsilon$$,

53 $$ \varepsilon > 0 $$

where, $$\left| {a_{x} } \right| \le ug$$.

**Figure 7:**
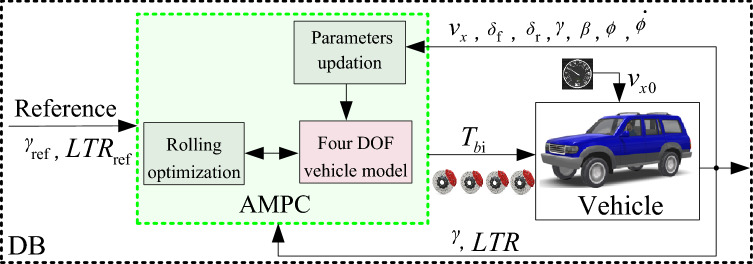
DB using AMPC.

The Block diagram of the DB using AMPC for rollover control is shown in Fig. 7.

The DB using AMPC takes *v*_*x*_, steering angle, and vehicle state of *γ, β, ϕ*, $\dot{\phi }$ as inputs to update the parameters of the internal four DOF vehicle model. The outputs of the AMPC is the braking forces of four wheels *F*_bi_ (i = 1, 2, 3, 4), and $T_{{{\text{bi}}}} = F_{{{\text{bi}}}} \cdot r_{w}$.

*Remark 3*: Different from the previous work^19,26^, which calculates the additional moment first, then allocates the braking moment on four wheels, the DB proposed in this article calculates the desired braking forces within the effective scope of the actuator and there is no need to distribute it to four wheels.

### Integrated steering and braking control

In this section, a novel integrated collision avoidance strategy for autonomous vehicles in an emergency based on steering and braking is designed.

The up-level decision-making layer is as follows: According to the vehicle status signal, the yaw rate *γ* and rollover index LTR are obtained. Then, they are compared with the *γ*_s (_ideal yaw rate_)_ and rollover threshold value *LTR*_s_. If the deviation of the actual and ideal yaw rates is less than ∆*γ*_s_, the ARS is not working, or the ARS is working. When the actual LTR is higher than *LTR*s, the DB is open. The collision avoidance strategy of the up-level decision-making layer is shown in Table 1.

**Table 1:** collision avoidance strategy of the up-level decision-making layer.

Control model	Selection conditions	4WS	ARS	DB
1	(∆*γ* < ∆*γ*_s_) and (LTR < LTR_s_)	Open	Close	Close
2	(∆*γ* > ∆*γ*_s_) and (LTR < LTR_s_)	Open	Open	Close
3	(∆*γ* > ∆*γ*_s_) and (LTR > LTR_s_)	Open	Open	Open

*4WS* Four-wheel steering, *ARS* Active rear steering, *DB* Differential braking.

**Figure 8:**
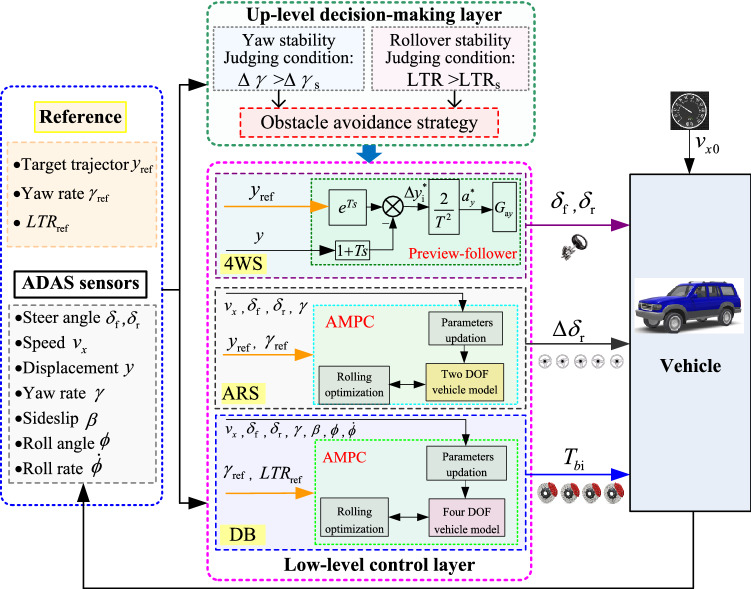
Flow chart of the integrated emergency collision avoidance decision-control.

The low-level is to control the vehicle according to the instructions generated by the up-level. The control strategy is integrating with 4 WS, ARS and DB by AMPC. The flow chart of the integrated emergency collision avoidance is shown in Fig. 8.

### Simulation results

In this section, the performance of the designed collision avoidance control strategy is evaluated by CarSim and MATLAB/Simulink co-simulation. The test vehicle is a CarSim *SUV* model of a SERES SF5, with the parameters listed in Table 2. To validate the performance of the integrated emergency collision avoidance strategy by 4 WS, ARS, and DB, a double lane change (DLC) maneuver and a “Sine” steering input at the speed of 80 km/h, 100 km/h, 120 km/h are performed in CarSim environment, and the Root Mean Square (RMS) values of path tracking error, yaw rate and, and roll angle are taken as the quantitative evaluation indexes to evaluate the performance of the integrated controller for collision avoidance in an emergency.

**Table 2:** Simulation parameters.

Parameters	Value
Sprung mass, total mass *m*_s_, *m*	2100, 2370 kg
Front, rear unsprung mass *m*_uf_, *m*_ur_	120, 150 kg
Front, rear axle distance to CG *l*_f_, *l*_r_	1.180, 1.695 m
Wheel track width of front, rear axle *t*_wf_, *t*_wr_	1.655, 1.650 m
Front, rear suspension roll stiffness *K*_*ϕ*f_, *K*_*ϕ*r_	92312, 89311 Nm/rad
CG height to ground *h*	0.720 m
CG height to roll center *h*_s_	0.340 m
Wheel roll radius *r*_w_	0.390 m
Yaw moment of inertia *I*_*z*_	2687 kg⋅m^2^
Roll moment of inertia *I*_*x*_	894.4 kg⋅m^2^
Roll damping coefficient C_*ϕ*_	5825 Nm·s/rad
Acceleration due to gravity *g*	9.81 m/s^2^

### Carsim SUV vehicle model

CarSim simulation software is used for the validation dynamic model and control strategy designed in this article. The simulation parameters are given in Table 2.

**Figure 9:**
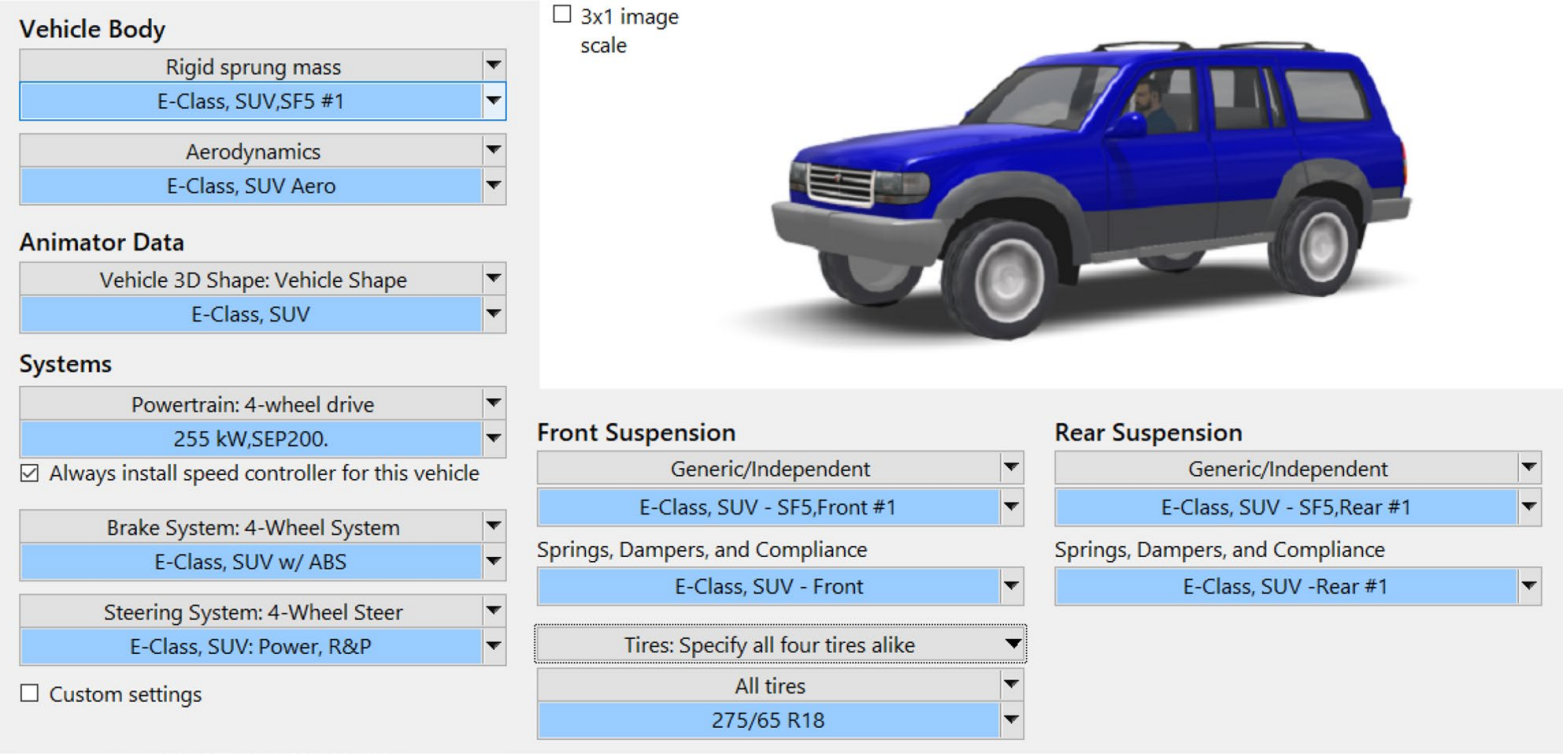
CarSim modeling interface.

To design the steering controller, the cornering stiffnesses of the front and rear axles *k*_f_ and *k*_r_ are set to 110367 N/rad and 70287 N/rad, respectively. A 275/65R18 radial pneumatic tire and generic front and rear independent suspensions are selected. According to Eq. (15), the gain coefficient of the four-wheel steering system *S*_1_ = -1, *S*_2_ = $- \frac{{\left( {1{ + }Kv_{x}^{2} } \right)l}}{{v_{x} }}$. Figure 9 shows a SUV simulated in CarSim.

### Co-simulation by steering control

**Figure 10:**
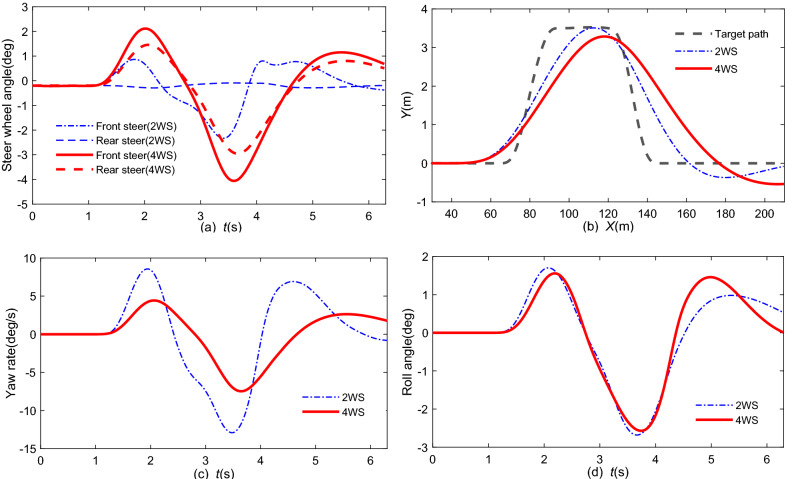
Simulation comparison with 4 WS under the DLC condition: (**a**) front, rear wheel steering angle; (**b**) lateral position; (**c**) yaw rate; (**d**) roll angle.

From the statistics of traffic crashes, changing lanes is very hazardous on busy highways. Therefore, a DLC is utilized to verify the proposed 4 WS system^19^. The initial speed v_x0_ is 120 km/h. The comparisons of the front and rear steering angles and driving state responses by 4 WS and 2 WS are shown in Fig. 10.

**Figure 11:**
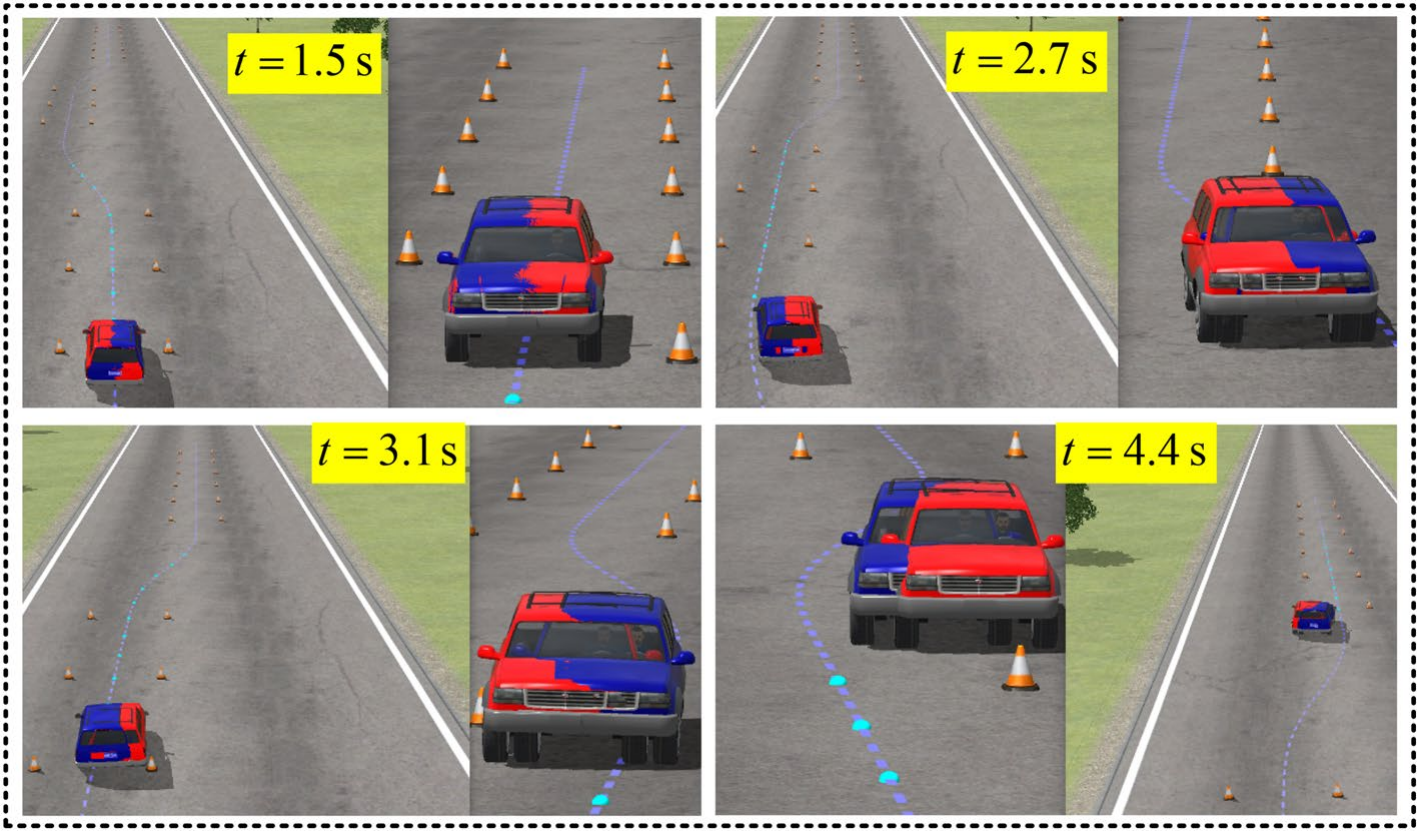
Dynamic visualization of collision avoidance (the blue and red cars are related to 2 WS and 4 WS, respectively).

The results given in Fig. 10c,d show that the maximum *γ* is reduced by − 46.04% at 3.5 s and that the roll angle is reduced by − 30.1% at 5 s by 4 WS. However, the front steer angle increased by 61.8% in the peak value at approximately 1.8 s in Fig. 10a, and the path tracking performance was also reduced in Fig. 10b. This means that the general 4 WS can help to improve the driving stability but will increase the front steer angle and path tracking error simultaneously. Figure 11 shows the 3D result of this scenario, where the blue and red cars represent 2 WS and 4 WS, respectively.

**Figure 12:**
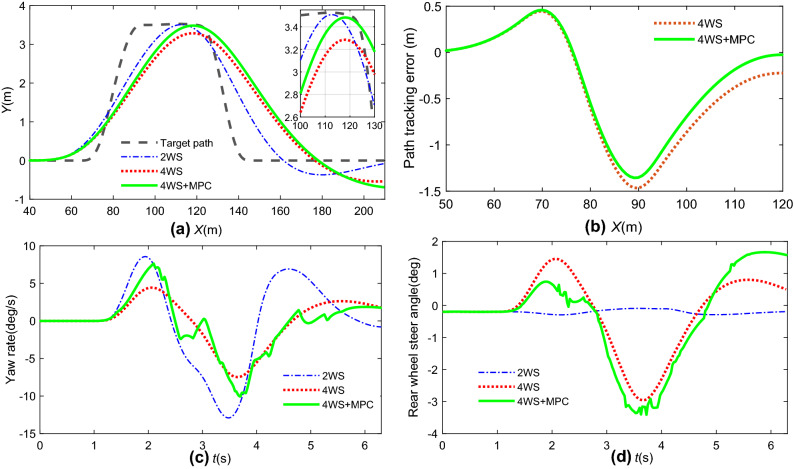
State response comparison by different controllers: (**a**) path tracking; (**b**) path tracking error; (**c**) yaw rate; (**d**) rear steering angle.

**Figure 13:**
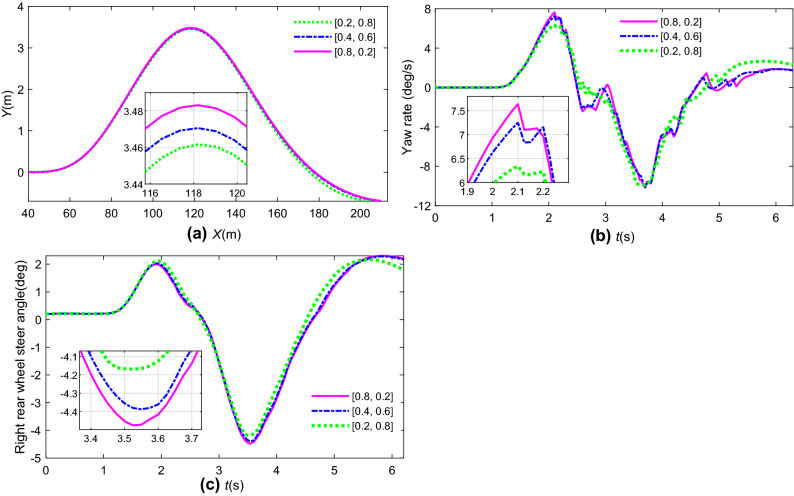
State response comparison at different weight matrices: (**a**) path tracking; (**b**) yaw rate; (**c**) rear steering angle.

The reference path is generated by a DLC for the tracking controller. The MPC simulation parameters were set as *N*_*p*_ = 20, *N*_*c*_ = 2, *r* = 1000, and *Q* = [2, 0.2]. *R* = [0, 1], *ε* = 1. The path tracking results, yaw stability index, and rear steering angle comparisons by MPC + 4 WS are shown in Fig. 12. The results given in Fig. 13 show the tracking performance and stability indices of 4 WS + MPC in different weight matrices of *Q* ([0.8 0.2], [0.4 0.6], [0.2 0.8]), respectively.

It can be seen from Fig. 12a,b that the peak value of the vehicle yaw rate was reduced by 30% by 4 WS + MPC at 3.5 s compared with 2 WS and that the path tracking performance was also enhanced compared with 4 WS. In addition, the MPC has a negative effect on rear wheel steering at 2 s for good accuracy of path tracking. In other words, 4 WS with an MPC controller can help to improve the driving stability and tracking performance simultaneously.

Figure 13b,c shows that the weight of the path tracking index increases with the error between the yaw rate and its reference values. For 4 WS + MPC with a high weight of lateral displacement, the steering angle of the rear wheel and yaw rate response should be larger than those of 4 WS + MPC with a low weight. 4 WS + MPC with a high weight of the yaw rate index can give better stability control performance because it can obtain more weight considerations in the process of optimization control.

**Figure 14:**
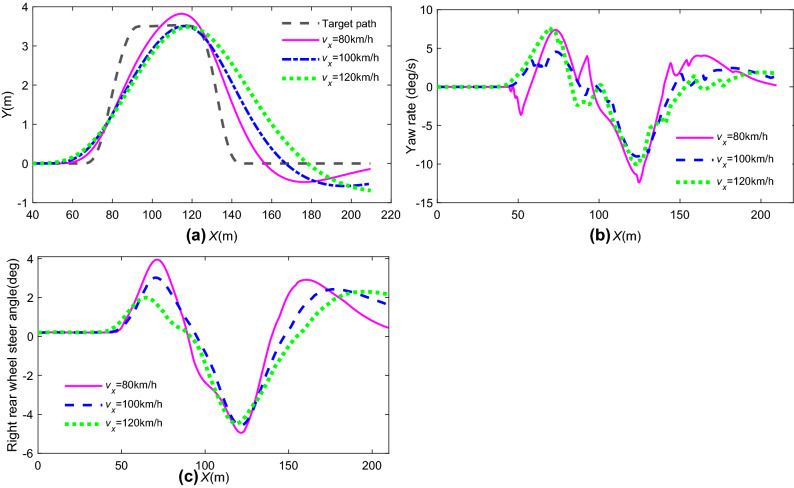
State response comparison at different longitudinal speeds: (**a**) path tracking; (**b**) yaw rate; (**c**) rear steering angle.

Figure 14 shows the vehicle tracking accuracy and stability indices of 4 WS + MPC at various velocities (80 km/h, 100 km/h, 120 km/h).

In Fig. 14, it is concluded that the peak value of the lateral station, yaw rate, and rear wheel steering angle changed obviously. When *v*_x0_ = 80 km/h, the tracking accuracy was good, but the stability index of the yaw worsened. When *v*_x0_ = 120 km/h, vehicle driving stability is best, but tracking accuracy worsens. In other words, the proposed path tracking control strategy based on MPC cannot adapt to vehicle speed changes completely.

**Figure 15:**
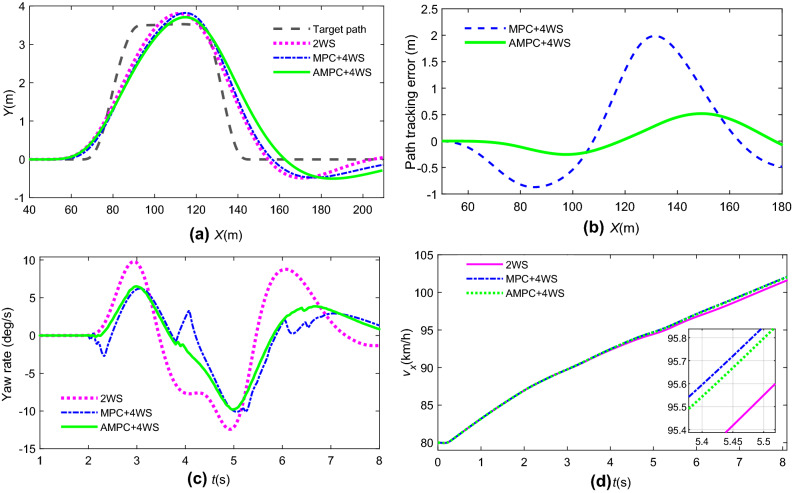
Path tracking and state response by different controllers: (**a**) path tracking; (**b**) path tracking error; (**c**) yaw rate; (**d**) vehicle longitudinal speed.

In Fig. 15, the vehicle yaw rate is reduced by more than 30% in peak value by AMPC + 4 WS compared with general vehicle (2 WS), and the AMPC + 4 WS is better to adapt to variable speed conditions under tracking collision avoidance compared with MPC + 4 WS controller.

### Co-simulation by braking control

**Figure 16:**
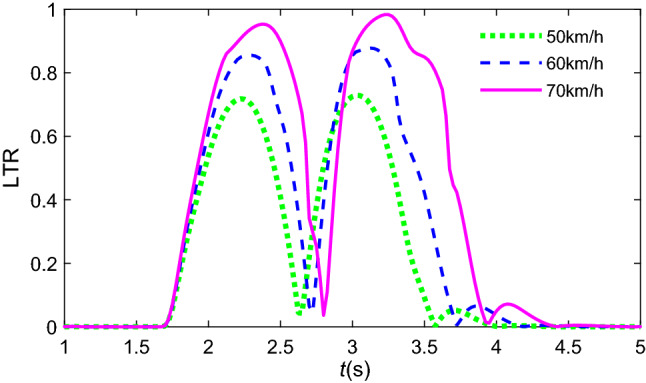
Transient response of LTR at different initial speeds.

To verify the effectiveness of the LTR by Eq. (48), the same “Sine” steering input is applied to the vehicle at different speeds, and Fig. 16 shows the transient response of the LTR at different initial speeds.

Figure 16 indicates that the rollover risk increases with increasing vehicle speed, especially vehicle speed over 70 km/h. The vehicle rollover index reaches its maximum threshold limit (LTR≈1) when avoiding obstacles with the same “Sine” steering input. The rollover status approaches *t* = 3.3 s, and LTR can estimate the point.

**Figure 17:**
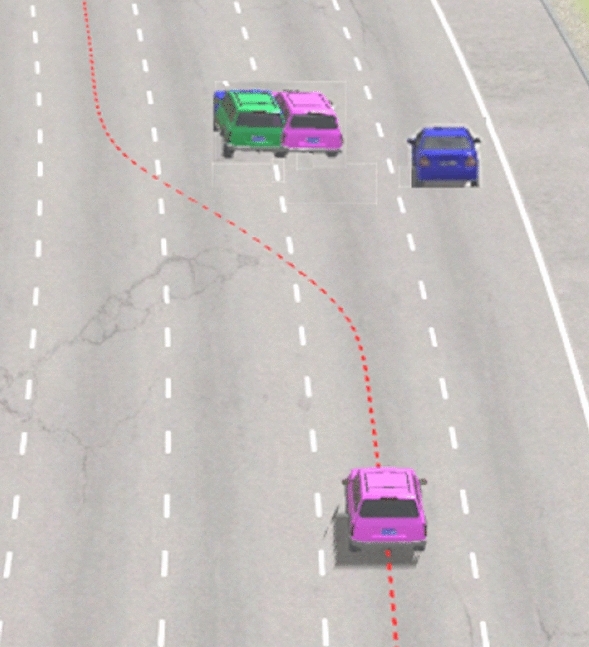
Dynamic visualization in highway emergency collision avoidance.

**Figure 18:**
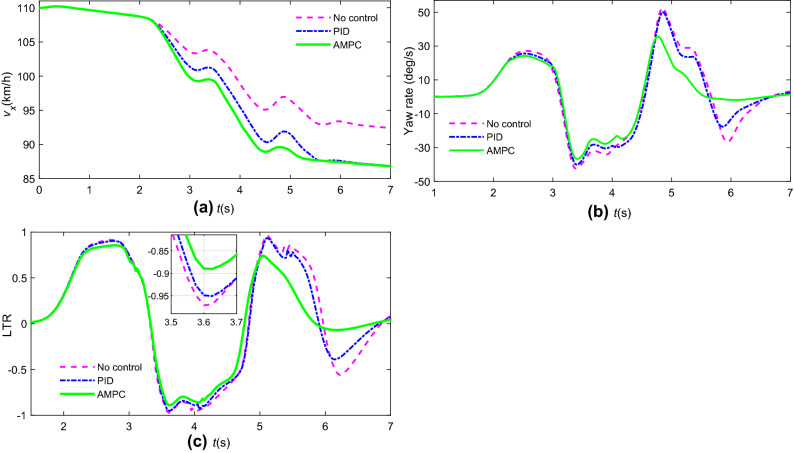
Stability index by different controllers: (**a**) vehicle speed; (**b**) yaw rate; (**c**) LTR.

**Figure 19:**
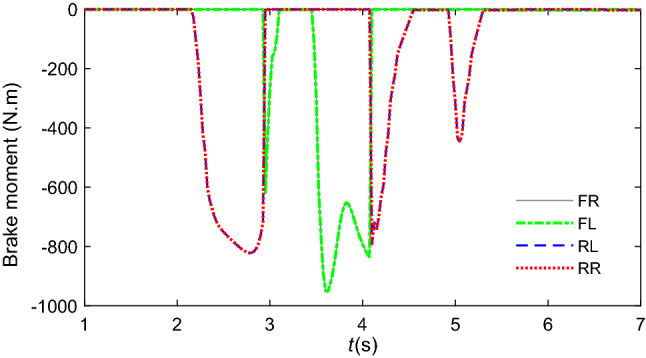
Active braking torque of 4 wheels by AMPC.

A traffic crash scenario occurring ahead of the vehicle on a highway is used to verify the rollover control based on AMPC, supposing that the 4 WS + ARS is not working. The vehicle needs to avoid obstacles immediately. The dynamic visualization is displayed in Fig. 17, and *v*_*x*0_ = 110 km/h. Figure 18 shows the stability index comparisons by AMPC and traditional PID. Figure 19 shows the differential braking torque of four wheels by AMPC.

Note that in Fig. 18b,c, the yaw rate peak values and LTR are decreased in the case of the anti-rollover control vehicle. However, the peak values of LTR by AMPC, PID, and no control are approximately 0.88, 0.94, and 0.98 respectively, which means that the proposed rollover controller can enhance the roll stability of the vehicle in emergency collision avoidance.

In Fig. 19, the rollover controller by AMPC generates a braking torque of 960 N·m to prevent rollover occurrence at 3.6 s as the increasing value of LTR (Fig. 18), and the vehicle speed also decreases rapidly which can prove that the braking controller has come into play.

### Co-simulation by integrated control

**Figure 20:**
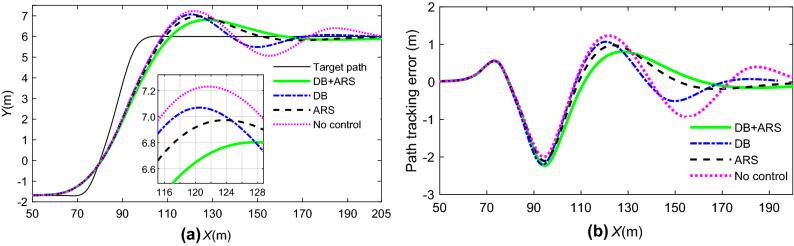
Vehicle tracking performance: (**a**)path tracking; (**b**) path tracking error.

**Figure 21:**
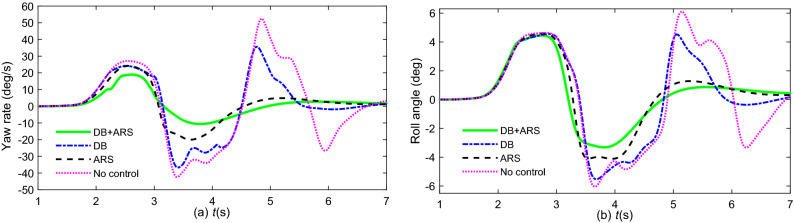
Vehicle driving stability: (**a**) yaw rate; (**b**) roll angle.

To verify the effectiveness of the integrated collision avoidance control based on AMPC, the dynamic visualization of collision avoidance is the same as in Fig. 17. The path tracking error of vehicles without stability control is the target of comparison. Figure 20 is the path tracking performance by different controllers. Figure 21 is the driving stability response of yaw and roll for emergency collision avoidance.

It is observed from Fig. 20 that the path tracking errors of a vehicle with ARS or DB are lower than those of the comparative group in different levels under emergency collision avoidance. The ARS and DB have the contribution to restrain the growth of lateral acceleration and keep the driving stability of the vehicle. Therefore, the tracking performance of DB + ARS is the best as it takes advantage of the DB and ARS.

Figure 21a shows that the yaw rate of the uncontrolled vehicle reaches 54 deg/s, which means that the vehicle is close to losing lateral stability. However, the vehicle controlled by DB + ARS and ARS can maintain lateral stability. In addition, according to Fig. 21b, compared with the vehicle with no control at 3.7 s, the peak value of the roll angle of the vehicle with DB + ARS and DB is reduced by 40% and 10%, respectively. In other words, the integrated controller can effectively prevent rollover under emergency conditions. Consequently, the vehicle with DB + ARS can perform steering and braking maneuvers to avoid collision in an emergency.

The Root Mean Square (RMS) values of simulation results by different control strategies are listed in Table 3.

**Table 3:** RMS values of different control strategies.

Values	DB + ARS	DB	ARS	No control
Path tracking error	0.6101 (m)	0.6774 (m)	0.6576 (m)	0.7278 (m)
Yaw rate	6.7412 (deg/s)	15.9064 (deg/s)	9.9851 (deg/s)	21.2249 (deg/s)
Roll angle	1.9370 (deg)	2.6834 (deg)	2.2089 (deg)	3.1365 (deg)

It shows that, compared with DB, and ARS, the RMS values with respect to tracking error, yaw rate and roll angle of DB + ARS are the smallest.

## Results discussion

Based on the above 4 groups of simulation results by 4WS^17^, ARS^19^, DB^22,26,27^, and DB + ARS, respectively. the following conclusions can be drawn.

- the general 4 WS can help to improve the driving stability but will increase the path tracking error, The ARS based on AMPC can give better stability control performance because it can obtain more weight considerations in the process of optimization control compared with 4WS.
- ARS and DB contribute to keeping the driving stability in an emergency by restraining the growth of lateral acceleration. However, the path tracking performance is been limited.
- The proposed DB + ARS can effectively prevent rollover, and improve the tracking performance as it takes advantage of the DB and ARS. However, the delay of the actuators does not take into account, thus the practical application in autonomous driving is limited.

## Conclusions and outlooks

An integrated collision avoidance strategy, composed of an up-level decision-making layer and a low-level controller layer, is proposed based on AMPC in this paper. The low-level is to control the vehicle according to the instructions generated by the up-level. The control strategy is integrating with 4 WS, ARS and DB by AMPC. Finally, the effectiveness of the proposed collision avoidance control strategy is validated by Carsim-Simulink co-simulation. The results are summarized as follows.

- For the design of ARS with high weight on lateral displacement, the rear steering angle and yaw rate responses are larger than those of controllers with low weight, and the ARS with a high weight of yaw rate index can obtain a better stability control performance.
- The designed ARS and DB work alone have limited effects on collision avoidance tracking performance and driving stability, and the collision avoidance control strategy for 4 WS autonomous vehicle based on AMPC is better adapted to variable speed compared with MPC.
- The 4 WS vehicle with DB + ARS can perform steering and braking maneuvers to avoid collision in an emergency. The performance of the proposed DB + ARS based on APMC for collision avoidance is better than that of the DB and ARS, and can reduce the peak value of yaw rate and roll angle by 40% under sufficient tracking accuracy, which means that the proposed strategy performed well in path tracking and driving stability.

Overall, the proposed integrated collision avoidance strategy can not only guarantee the path tracking accuracy of the vehicle but also enhance driving stability under emergency conditions. However, the individual differences of the drivers and the delay of the actuators, are two key questions for the collision avoidance control strategy in actual situations, which do not take into account in this paper. Therefore, personalized motion control strategy and the robustness of the controller for the integrated collision avoidance system need further study. In addition, experimentation will be followed in the next investigation to test the control scheme.

## Data Availability

The datasets used and/or analysed during the current study available from the corresponding author on reasonable request.
